# Profiles of Extra‐Task Workload Among Clinical Nurses and Associations With Narrative Cognitive Ability: A Latent Profile Analysis

**DOI:** 10.1155/jonm/7831111

**Published:** 2026-09-22

**Authors:** Xue Li, Weihong Yang, Ling Guo, Ruijuan Fan, Hua Peng, Fengpei Zhang

**Affiliations:** ^1^ Department of Nursing, The First Affiliated Hospital of Henan Medical University, Xinxiang, Henan, China; ^2^ School of Nursing, Henan Medical University, Xinxiang, Henan, China; ^3^ Department of Thoracic Surgery Ward, The First Affiliated Hospital of Henan Medical University, Xinxiang, Henan, China; ^4^ Department of Oncology Ward, The First Affiliated Hospital of Henan Medical University, Xinxiang, Henan, China

**Keywords:** clinical nurses, extra-task workload, latent profile analysis, narrative cognitive ability, nursing management

## Abstract

**Background:**

In addition to providing direct care, clinical nurses often undertake teaching, research, quality improvement and care coordination responsibilities. However, whether these extra‐task demands form distinct workload patterns, and how such patterns relate to narrative cognitive ability, remains unclear.

**Aim:**

To identify distinct extra‐task workload patterns among clinical nurses and compare narrative cognitive ability across the identified profiles.

**Methods:**

A cross‐sectional survey was conducted among 1081 clinical nurses at a tertiary general hospital in Henan Province, China, between March and April 2025. Data were collected using a demographic questionnaire, the Clinical Nurse Extra‐Task Workload Scale and the Narrative Cognitive Ability Scale. Latent profile analysis was performed to identify extra‐task workload profiles, and covariate‐adjusted Bolck–Croon–Hagenaars (BCH) analysis was used to compare narrative cognitive ability across the identified profiles.

**Results:**

Four profiles were identified: moderate demands–minimal constraints (12.40%), high demands–high constraints (18.59%), moderate demands–low constraints (25.16%) and elevated demands–moderate constraints (43.85%). Profile membership differed according to several demographic and work‐related characteristics, including night‐shift frequency, work‐related mood, leisure activities and narrative training experience. After covariate adjustment, significant differences remained in overall narrative cognitive ability, life and health narrative awareness and professional narrative thinking. Nurses in the moderate demands–minimal constraints profile demonstrated the highest levels of narrative cognitive ability.

**Conclusion:**

Extra‐task workload among clinical nurses exhibited distinct profile heterogeneity. Different combinations of work environment demands and behavioural constraints were associated with narrative cognitive ability.

**Implications for Nursing Management:**

Nursing managers should adopt profile‐informed strategies rather than uniform workload interventions for clinical nurses. Identifying nurses with different extra‐task workload profiles may help guide targeted management measures, including optimisation of work demands, clarification of role expectations and tailored narrative training and professional support programmes.

## 1. Introduction

Nurses represent the largest professional group in the healthcare workforce and play a critical role in ensuring quality of care, patient safety and continuity of healthcare delivery. According to the State of the World’s Nursing 2025 report published by the World Health Organization (WHO), the global nursing workforce increased from 27.9 million in 2018 to 29.8 million in 2023. However, a shortage of approximately 5.8 million nurses persisted worldwide, alongside substantial inequities in the distribution of nursing resources [1]. In China, the nursing workforce continued to expand, reaching 5.855 million registered nurses, equivalent to 4.16 registered nurses per 1000 population, in 2024. During the same period, healthcare utilisation remained high, with 10.15 billion visits to healthcare institutions and 312 million hospital admissions nationwide [2]. As healthcare demand has increased and organisational expectations have evolved, clinical nurses’ responsibilities have expanded beyond direct patient care to include a growing range of non–direct‐care activities [3, 4]. Understanding clinical nurses’ workload in contemporary healthcare is therefore essential for optimising nursing workforce management.

Previous research on nursing workload has primarily focused on overall workload, clinical care burden and demands associated with direct nursing practice, with limited attention to the additional workload arising from responsibilities beyond routine nursing duties [5, 6]. As nursing service models continue to evolve, clinical nurses are increasingly expected to undertake teaching, research, quality improvement and organisational coordination activities [7, 8]. Although these responsibilities contribute to service delivery and organisational functioning, their cumulative burden may increase job demands and consume nurses’ limited time and cognitive resources [9, 10]. Extra‐task workload refers to the workload experienced by clinical nurses when undertaking responsibilities beyond routine nursing duties. It comprises two dimensions: work environment demands and individual behavioural constraints [11]. Work environment demands reflect additional requirements arising from organisational arrangements, role expansion and workplace expectations, whereas individual behavioural constraints describe the extent to which these responsibilities restrict nurses’ perceived control and behavioural flexibility. Because clinical nurses differ in both task demands and resource availability, extra‐task workload may manifest in distinct patterns across individuals. However, existing studies have mainly examined overall levels of extra‐task workload, leaving its underlying heterogeneity insufficiently understood.

The job demands–resources (JD–R) theory provides a useful framework for understanding how job demands influence nurses’ professional functioning. The theory proposes that excessive or sustained job demands consume individuals’ limited resources, which may subsequently affect their psychological states and work‐related behaviours [12, 13]. Narrative cognitive ability, an important component of nursing competence, refers to nurses’ capacity to understand, interpret and respond to patients’ experiences through contextual understanding and reflective processing [14]. Because developing and applying narrative cognitive ability require sustained attention and cognitive engagement [15, 16], higher extra‐task workload may be associated with lower levels of narrative cognitive ability.

Previous studies of nurses’ workload have largely adopted variable‐centred approaches to examine average associations between workload and occupational outcomes [17, 18]. Although this approach has provided valuable insights, it assumes relative homogeneity among individuals and may mask meaningful subgroup differences arising from distinct workload combinations [19]. For extra‐task workload, focussing solely on overall levels may fail to identify clinical nurses experiencing different combinations of external demands and behavioural constraints. Latent profile analysis (LPA) is a person‐centred method that identifies subgroups with similar patterns across multiple continuous indicators [20, 21]. It can capture such heterogeneity and reveal distinct profiles within complex occupational settings. Although LPA has been increasingly used in nursing research to identify heterogeneous patterns of psychological states, occupational characteristics and work experiences [22, 23], few studies have examined latent profiles of extra‐task workload among clinical nurses or their association with narrative cognitive ability.

Therefore, guided by JD–R theory, this study employed LPA to identify latent profiles of extra‐task workload among clinical nurses and examine differences in narrative cognitive ability across the identified profiles.

The following hypotheses were proposed:

 H1: Clinical nurses exhibit distinct latent profiles of extra‐task workload.
 H2: Clinical nurses with different extra‐task workload profiles demonstrate different levels of narrative cognitive ability.

## 2. Methods

### 2.1. Design

This cross‐sectional study adhered to the Strengthening the Reporting of Observational Studies in Epidemiology (STROBE) guidelines for cross‐sectional studies.

### 2.2. Participants

Clinical nurses from a tertiary Grade A general hospital in Henan Province, China, were recruited through convenience sampling between March and April 2025. Nurses were eligible if they were registered nurses, had at least 1 year of clinical experience and provided informed consent to participate. Nurses were excluded if they were absent during data collection, worked in nonclinical posts or were interns or residents in training.

### 2.3. Sample Size

Because LPA was the primary analytical method, sample‐size planning was guided by methodological considerations from mixture‐modelling studies, with a multiple linear regression model specified in G∗Power 3.1 as an auxiliary reference. Assuming an effect size of *f* ^2^ = 0.10, *α* = 0.05, a statistical power of 0.95 and 12 predictors, the estimated minimum sample size was 270. Allowing for 20% potentially invalid responses increased the required sample size to 338. Considering methodological guidance on profile estimation and previous empirical applications of LPA [24, 25], a pragmatic target of at least 500 participants was considered appropriate. Ultimately, 1081 valid questionnaires were included in the analysis.

### 2.4. Measurements

#### 2.4.1. Demographic Characteristics

Demographic and work‐related characteristics were collected using a structured questionnaire developed for this study. The questionnaire included 10 variables: gender, age, years of clinical experience, professional title, administrative position, educational level, monthly night‐shift frequency, work‐related mood, participation in leisure activities and previous narrative nursing training.

#### 2.4.2. Clinical Nurse Extra‐Task Workload Scale

Extra‐task workload was assessed using the Clinical Nurse Extra‐Task Workload Scale developed by Yang et al. [11]. The scale, developed based on the job demand–control model, comprises 12 items across two dimensions: work environment demands and individual behavioural constraints. Items are rated on a 5‐point Likert scale, with higher scores indicating greater extra‐task workload. The original validation study, conducted using an independent sample of clinical nurses, demonstrated satisfactory psychometric properties, including internal consistency (Cronbach’s *α* = 0.950 for the total scale) and evidence of construct validity. In the present study, Cronbach’s *α* coefficients were 0.664 for work environment demands, 0.919 for individual behavioural constraints and 0.863 for the total scale.

#### 2.4.3. Narrative Cognitive Ability Scale

Narrative cognitive ability was assessed using the Narrative Cognitive Ability Scale, a subscale of the Chinese Medical Practitioners’ Narrative Competencies Scale developed by Yang et al. [14]. The scale comprises 23 items across two dimensions: life and health narrative awareness and professional narrative thinking. Items are rated on a 4‐point Likert scale ranging from 1 (‘*completely disagree*’) to 4 (‘*completely agree*’), with higher scores indicating higher levels of narrative cognitive ability. The original validation study, conducted among medical professionals, demonstrated satisfactory psychometric properties through exploratory and confirmatory factor analyses, with internal consistency (Cronbach’s *α* = 0.98 for the total scale). In the present study, Cronbach’s *α* coefficients were 0.936 for life and health narrative awareness, 0.983 for professional narrative thinking and 0.979 for the total scale.

#### 2.4.4. Data Collection

After institutional approval was obtained, an anonymous online survey was administered through Wenjuanxing. Eligible nurses received the survey link through departmental communication channels and provided electronic informed consent before completing the questionnaire. To reduce potential common method bias, participants were assured that the survey was anonymous, confidential and used solely for research purposes. Each participant could submit the questionnaire once. After collection, the dataset was checked for completeness and response quality. Questionnaires with implausible response patterns were excluded. No missing values were identified among the questionnaires retained for analysis; therefore, no missing‐data imputation was performed. Of the 1242 eligible nurses invited to participate, 1081 returned valid questionnaires, corresponding to an effective response rate of 87.04%.

### 2.5. Data Analysis

Data were analysed using SPSS 25.0 and Mplus 8.0. Continuous variables were summarised as means and standard deviations, whereas categorical variables were described using frequencies and percentages. Group differences across profiles were examined using the chi‐square test, Fisher’s exact test or the Kruskal–Wallis H test according to variable type and distribution.

LPA was conducted using Mplus 8.0 to identify distinct patterns of extra‐task workload. In the primary analysis, the 12 items of the Clinical Nurse Extra‐Task Workload Scale were used as continuous indicators. Models with increasing numbers of profiles were estimated sequentially and evaluated using the Akaike information criterion (AIC), Bayesian information criterion (BIC), adjusted Bayesian information criterion (aBIC), entropy, Lo–Mendell–Rubin (LMR) likelihood ratio test, bootstrapped likelihood ratio test (BLRT) and profile size. The optimal model was selected based on statistical fit, classification quality, interpretability and profile stability. A sensitivity analysis was subsequently conducted using the summed scores of the two subscales, work environment demands and individual behavioural constraints, as indicators. Consistency between the item‐level and dimension‐level solutions was evaluated based on profile patterns, proportions and classification agreement, with the latter quantified using the percentage of identical modal classifications, Cohen’s kappa and the adjusted Rand index.

Differences in narrative cognitive ability across latent profiles were examined using the BCH three‐step approach while accounting for classification uncertainty. Covariates were incorporated during the BCH adjustment step to reduce potential confounding, and adjusted mean differences between profiles were estimated. Cohen’s d was calculated using the adjusted mean difference and the corresponding model residual variance to quantify the magnitude of pairwise differences. Statistical significance was set at *p* < 0.05.

### 2.6. Ethical Consideration

Ethical approval for this study was obtained from the Ethics Committee of the First Affiliated Hospital of Henan Medical University (approval number: EC‐025‐591). All participants provided informed consent before participation. The survey was conducted anonymously, and participant confidentiality was maintained throughout the study.

## 3. Results

### 3.1. Assessment of Common Method Bias

To assess the potential influence of common method bias, Harman’s single‐factor test was conducted using all measurement items [26]. Five factors with eigenvalues greater than 1 were extracted, and the first unrotated factor accounted for 48.79% of the total variance. As this proportion was below the commonly accepted threshold of 50%, common method bias was considered unlikely to have substantially influenced the study findings.

### 3.2. Participant Characteristics

Participants were aged between 22 and 55 years, with a mean age of 34.51 ± 6.44 years. Most participants were female (96.7%) and did not hold administrative positions (94.6%). Regarding clinical experience, 45.9% had 1–10 years of experience. Most participants held the title of nurse supervisor (65.4%). For monthly night‐shift frequency, 28.1% reported 9–12 shifts per month. Most participants held a bachelor’s degree (92.1%), and 32.7% reported a positive work‐related mood. Additionally, 2.7% reported frequent participation in leisure activities, and 10.9% reported often participating in narrative nursing training. Detailed demographic and work‐related characteristics are presented in Table 1.

**TABLE 1:** Univariate analysis of general demographic characteristics and 4 latent classes of extra‐task workload among clinical nurses (*N* = 1081, *n* [%]).

Characteristics	Overall (*N* = 1081) (%)	Classification of latent profiles				Test statistic	*p*
		Class 1 (%) (*n* = 134)	Class 2 (%) (*n* = 201)	Class 3 (%) (*n* = 272)	Class 4 (%) (*n* = 474)		
Gender						Fisher’s exact = 3.223	0.358
Male	36 (3.3)	3 (8.3)	9 (25.0)	12 (33.3)	12 (33.3)		
Female	1045 (96.7)	131 (12.5)	192 (18.4)	260 (24.9)	462 (44.2)		
Age (years)						*H* = 2.237	0.525
≤ 30	312 (28.9)	34 (10.9)	57 (18.3)	88 (28.2)	133 (42.6)		
31–40	604 (55.9)	74 (12.3)	119 (19.7)	134 (22.2)	277 (45.9)		
41–50	135 (12.5)	19 (14.1)	21 (15.6)	36 (26.7)	59 (43.7)		
> 50	30 (2.8)	7 (23.3)	4 (13.3)	14 (46.7)	5 (16.7)		
Years of clinical experience						*H* = 2.024	0.567
1–10	496 (45.9)	55 (11.1)	87 (17.5)	138 (27.8)	216 (43.5)		
11–20	488 (45.1)	68 (13.9)	95 (19.5)	98 (20.1)	227 (46.5)		
21–30	66 (6.1)	4 (6.1)	14 (21.2)	24 (36.4)	24 (36.4)		
> 30	31 (2.9)	7 (22.6)	5 (16.1)	12 (38.7)	7 (22.6)		
Professional title						*H* = 3.259	0.353
Nurse	75 (6.9)	10 (13.3)	10 (13.3)	32 (42.7)	23 (30.7)		
Nurse practitioner	280 (25.9)	35 (12.5)	51 (18.2)	67 (23.9)	127 (45.4)		
Nurse supervisor	707 (65.4)	88 (12.4)	136 (19.2)	165 (23.3)	318 (45.0)		
Cochief superintendent nurse	19 (1.8)	1 (5.3)	4 (21.1)	8 (42.1)	6 (31.6)		
Administrative position						*χ* ^2^ = 2.465	0.482
No	1023 (94.6)	123 (12.0)	191 (18.7)	258 (25.2)	451 (44.1)		
Yes	58 (5.4)	11 (19.0)	10 (17.2)	14 (24.1)	23 (39.7)		
Education level						*H* = 6.135	0.105
Associate degree	55 (5.1)	9 (16.4)	8 (14.5)	21 (38.2)	17 (30.9)		
Bachelor’s degree	996 (92.1)	121 (12.1)	187 (18.8)	245 (24.6)	443 (44.5)		
Master’s degree	30 (2.8)	4 (13.3)	6 (20.0)	6 (20.0)	14 (46.7)		
Monthly night‐shift frequency						*H* = 15.610	0.001
0	254 (23.5)	43 (16.9)	36 (14.2)	66 (26.0)	109 (42.9)		
1–4 days/month	117 (10.8)	18 (15.4)	22 (18.8)	34 (29.1)	43 (36.8)		
5–8 days/month	194 (17.9)	19 (9.8)	32 (16.5)	51 (26.3)	92 (47.4)		
9–12 days/month	304 (28.1)	38 (12.5)	60 (19.7)	78 (25.7)	128 (42.1)		
> 12 days/month	212 (19.6)	16 (7.5)	51 (24.1)	43 (20.3)	102 (48.1)		
Work‐related mood						*H* = 203.816	< 0.001
Very happy	101 (9.3)	44 (43.6)	7 (6.9)	31 (30.7)	19 (18.8)		
Relatively happy	353 (32.7)	62 (17.6)	29 (8.2)	122 (34.6)	140 (39.7)		
Generally	533 (49.3)	27 (5.1)	116 (21.8)	113 (21.2)	277 (52.0)		
Unpleasant	71 (6.6)	0 (0.0)	39 (54.9)	4 (5.6)	28 (39.4)		
Very unpleasant	23 (2.1)	1 (4.3)	10 (43.5)	2 (8.7)	10 (43.5)		
Participation in leisure activities						*H* = 41.702	< 0.001
Never participate	383 (35.4)	40 (10.4)	104 (27.2)	71 (18.5)	168 (43.9)		
Occasional participate	560 (51.8)	65 (11.6)	80 (14.3)	151 (27.0)	264 (47.1)		
Sometimes participate	109 (10.1)	21 (19.3)	12 (11.0)	41 (37.6)	35 (32.1)		
Often participate	29 (2.7)	8 (27.6)	5 (17.2)	9 (31.0)	7 (21.4)		
Previous narrative training experience						*H* = 33.667	< 0.001
Often participation	118 (10.9)	27 (22.9)	14 (11.9)	34 (28.8)	43 (36.4)		
Sometimes participation	411 (38.0)	56 (13.6)	63 (15.3)	107 (26.0)	185 (45.0)		
Occasional participate	420 (38.9)	45 (10.7)	79 (18.8)	106 (25.2)	190 (45.2)		
Never participate	132 (12.2)	6 (4.5)	45 (34.1)	25 (18.9)	56 (42.4)		

*Note:* *χ* ^2^, chi‐square test; *H*, Kruskal–Wallis *H* test; Fisher’s exact, Fisher’s exact test.

### 3.3. Descriptive Statistics of Main Variables

The descriptive statistics of the main study variables are presented in Table 2. The mean total score for extra‐task workload was 35.40 ± 7.20, with mean subscale scores of 19.18 ± 3.32 for work environment demands and 16.22 ± 5.02 for individual behavioural constraints. The mean total narrative cognitive ability score was 74.28 ± 9.55, with mean subscale scores of 28.51 ± 3.40 for life and health narrative awareness and 45.77 ± 6.09 for professional narrative thinking.

**TABLE 2:** Participants’ extra‐task workload and narrative cognitive ability score.

Variables	The total score mean (SD)	The average score mean (SD)
Work environment demands	19.18 (3.32)	3.20 (0.55)
Individual behavioural constraints	16.22 (5.02)	2.70 (0.84)
Total clinical nurse extra‐task workload score	35.40 (7.20)	2.95 (0.60)
Life and health narrative awareness	28.51 (3.40)	3.17 (0.43)
Professional narrative thinking	45.77 (6.09)	3.27 (0.44)
Total narrative cognitive ability score	74.28 (9.55)	3.23 (0.42)

### 3.4. LPA of Extra‐Task Workload

In the primary item‐level analysis, latent profile models with one to five classes were estimated using the 12 item scores of the Clinical Nurse Extra‐Task Workload Scale as continuous indicators. The five‐class model failed to converge and was excluded from further consideration, possibly due to increased model complexity and unstable parameter estimation [27]. The four‐class solution showed the relative model fit, with the lowest AIC, BIC and aBIC values and satisfactory classification accuracy (entropy = 0.981). The LMR and BLRT supported the four‐class solution over the three‐class solution (*p* < 0.05), and all profile sizes exceeded 10%, indicating adequate profile stability. Therefore, the four‐class model was selected as the optimal solution (Table 3).

**TABLE 3:** Model fit indices for the primary item‐level and dimension‐level sensitivity latent profile analyses (*N* = 1081).

Analysis	Model	*K*	Log (*L*)	AIC	BIC	aBIC	Entropy	LMR	BLRT	Class probability
Primary item‐level LPA	1‐class	24	−17672.352	35392.704	35512.359	35436.130	—	—	—	—
	2‐class	37	−15815.874	31705.748	31890.217	31772.697	0.949	< 0.001	< 0.001	0.384/0.616
	3‐class	50	−14852.091	29804.182	30053.464	29894.653	0.955	< 0.001	< 0.001	0.343/0.472/0.185
	4‐class	63	−14199.361	28524.723	28838.818	28638.717	0.981	0.004	< 0.001	0.124/0.186/0.252/0.438
	5‐class	—	Did not converge	—	—	—	—	—	—	—

Dimension‐level sensitivity LPA	1‐class	4	−6107.630	12223.260	12243.202	12230.497	—	—	—	1.000
	2‐class	7	−5993.416	12000.833	12035.732	12013.499	0.558	< 0.001	< 0.001	0.659/0.341
	3‐class	10	−5914.931	11849.862	11899.719	11867.957	0.788	< 0.001	< 0.001	0.548/0.281/0.171
	4‐class	13	−5840.290	11706.581	11771.394	11730.103	0.898	< 0.001	< 0.001	0.460/0.253/0.116/0.171
	5‐class	16	−5815.115	11662.231	11742.001	11691.182	0.905	< 0.001	< 0.001	0.260/0.116/0.149/0.032/0.443

*Note:* The primary item‐level latent profile analysis (LPA) used the 12 item scores of the Clinical Nurse Extra‐Task Workload Scale. The dimension‐level sensitivity LPA used the summed scores for work environment demands (Items 1–6) and individual behavioural constraints (Items 7–12). *K* = number of free parameters; Log (*L*) = log‐likelihood; aBIC = sample‐size–adjusted Bayesian information criterion; LMR = Lo–Mendell–Rubin adjusted likelihood ratio test; BLRT = parametric bootstrapped likelihood ratio test; Entropy = classification accuracy index. Class proportions are presented in the class order returned by Mplus; class numbering is model‐specific and should not be compared directly across models. Bold shading indicates the retained four‐class solutions.

Abbreviations: AIC, Akaike information criterion; BIC, Bayesian information criterion.

**FIGURE 1:**
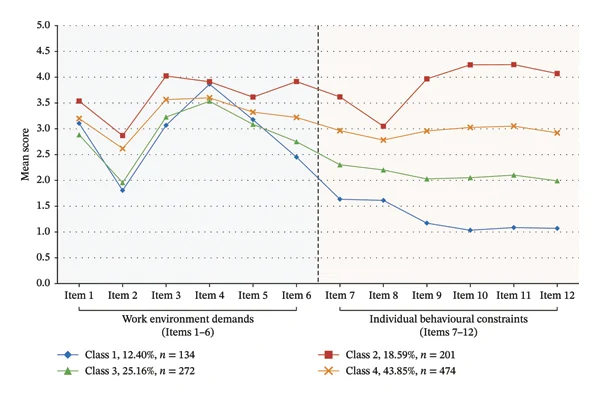
Characteristic distributions of four latent profiles of clinical nurses’ extra‐task workload.

Based on the response patterns across the two dimensions of extra‐task workload (work environment demands and individual behavioural constraints), four latent profiles were identified: Class 1, moderate demands–minimal constraints (12.40%); Class 2, high demands–high constraints (18.59%); Class 3, moderate demands–low constraints (25.16%); and Class 4, elevated demands–moderate constraints (43.85%). Figure 1 represents the response patterns for each latent profile.

To examine the robustness of the four‐profile solution, a dimension‐level sensitivity LPA was conducted using the summed scores of work environment demands and individual behavioural constraints as indicators. The four‐class solution showed good classification accuracy (entropy = 0.898) and yielded profile patterns and proportions broadly consistent with those identified in the primary item‐level analysis (11.56%, 17.11%, 25.35% and 45.98%). Although the five‐class model yielded lower information‐criterion values, it produced a small class comprising only 3.24% of the sample; therefore, the four‐class solution was retained considering profile size, parsimony and interpretability. After profile alignment, 88.7% of participants received the same modal classification across the two analyses (Cohen’s kappa = 0.836; adjusted Rand index = 0.711), further supporting the robustness of the four‐profile solution (Table 3).

### 3.5. Differences in Narrative Cognitive Ability Across Latent Profiles

After covariate adjustment, significant differences remained in life and health narrative awareness (Wald *χ* ^2^ = 79.749, *p* < 0.001), professional narrative thinking (Wald *χ* ^2^ = 97.423, *p* < 0.001) and total narrative cognitive ability score (Wald *χ* ^2^ = 100.449, *p* < 0.001). Class 1 had the highest adjusted estimated means scores across all three outcomes, followed by Class 3, whereas Classes 2 and 4 had relatively lower adjusted estimated means. Detailed results are presented in Table 4.

**TABLE 4:** Adjusted differences in narrative cognitive ability among nurses with different latent profiles of extra‐task workload using the BCH method (*N* = 1081).

Latent profile	*n* (%)	Life and health narrative awareness	Professional narrative thinking	Total narrative cognitive ability score
		Est. mean (SE)	Est. mean (SE)	Est. mean (SE)
Moderate demands–minimal constraints (Class 1)	134 (12.40)	31.65 (0.41)	50.66 (0.58)	82.31 (0.93)
High demands–high constraints (Class 2)	201 (18.59)	27.63 (0.25)^a^	44.96 (0.39)^a^	72.59 (0.58)^a^
Moderate demands–low constraints (Class 3)	272 (25.16)	28.85 (0.25)^ab^	46.52 (0.39)^ab^	75.36 (0.62)^ab^
Elevated demands–moderate constraints (Class 4)	474 (43.85)	27.80 (0.15)^ac^	44.32 (0.24)^ac^	72.12 (0.37)^ac^
Wald *χ* ^2^		79.749	97.423	100.449
*p*		< 0.001	< 0.001	< 0.001

*Note:* Bolck–Croon–Hagenaars method; Est. Mean = covariate‐adjusted model‐estimated marginal mean. Adjusted for monthly night‐shift frequency, work‐related mood, participation in leisure activities and previous narrative training experience.

Abbreviation: SE, standard error.

^a^ *p* < 0.05 compared with Class 1.

^b^ *p* < 0.05 compared with Class 2.

^c^ *p* < 0.05 compared with Class 3.

Pairwise comparisons further indicated that Class 1 had significantly higher scores than Classes 2, 3 and 4 (*p* < 0.001), with moderate‐to‐large effect sizes (|Cohen’s *d*| = 0.764–1.205). Class 3 also scored significantly higher than Classes 2 and 4 (*p* < 0.05), with small effect sizes (|Cohen’s *d*| = 0.287–0.405). No significant differences were observed between Classes 2 and 4, with negligible effect sizes (|Cohen’s *d*| = 0.050–0.118) (Table 5).

**TABLE 5:** Pairwise comparisons and standardised effect sizes for narrative cognitive ability across latent profiles using the covariate‐adjusted BCH method.

Outcome	Comparison	Adjusted mean difference	Cohen’s *d*	Wald chi‐square	*p*
Life and health narrative awareness	Class 1 vs. Class 2	4.021	1.147	66.260	< 0.001
	Class 1 vs. Class 3	2.798	0.798	34.810	< 0.001
	Class 1 vs. Class 4	3.845	1.097	72.795	< 0.001
	Class 2 vs. Class 3	−1.222	−0.349	11.431	0.001
	Class 2 vs. Class 4	−0.176	−0.050	0.391	0.532
	Class 3 vs. Class 4	1.047	0.299	12.166	< 0.001

Professional narrative thinking	Class 1 vs. Class 2	5.696	1.051	61.059	< 0.001
	Class 1 vs. Class 3	4.141	0.764	35.736	< 0.001
	Class 1 vs. Class 4	6.335	1.169	92.833	< 0.001
	Class 2 vs. Class 3	−1.555	−0.287	7.701	0.006
	Class 2 vs. Class 4	0.640	0.118	2.140	0.143
	Class 3 vs. Class 4	2.194	0.405	21.977	< 0.001

Total narrative cognitive ability score	Class 1 vs. Class 2	9.716	1.150	72.216	< 0.001
	Class 1 vs. Class 3	6.939	0.821	39.464	< 0.001
	Class 1 vs. Class 4	10.180	1.205	95.180	< 0.001
	Class 2 vs. Class 3	−2.777	−0.329	10.189	0.001
	Class 2 vs. Class 4	0.464	0.055	0.483	0.487
	Class 3 vs. Class 4	3.241	0.383	19.466	< 0.001

*Note:* Adjusted for monthly night‐shift frequency, work‐related mood, participation in leisure activities and previous narrative training experience.

### 3.6. Associations of Covariates With Narrative Cognitive Ability in the BCH Model

After covariate adjustment, work‐related mood and narrative training experience were significantly associated with narrative cognitive ability outcomes, whereas monthly night‐shift frequency and participation in leisure activities were not. Detailed results are presented in Table 6.

**TABLE 6:** Associations of covariates in the adjusted BCH models.

Covariate	Comparison	Life and health narrative awareness	Professional narrative thinking	Total narrative cognitive ability score
		*B* (SE), *p*	*B* (SE), *p*	*B* (SE), *p*
Monthly night‐shift frequency	1–4 days/month vs. 0	0.064 (0.403), *p* = 0.874	−0.579 (0.609), *p* = 0.342	−0.515 (0.978), *p* = 0.598
	5–8 days/month vs. 0	0.346 (0.347), *p* = 0.318	0.475 (0.532), *p* = 0.371	0.822 (0.826), *p* = 0.320
	9–12 days/month vs. 0	0.403 (0.312), *p* = 0.198	0.113 (0.482), *p* = 0.815	0.516 (0.755), *p* = 0.495
	> 12 days/month vs. 0	0.053 (0.324), *p* = 0.869	0.455 (0.521), *p* = 0.382	0.508 (0.790), *p* = 0.520

Work‐related mood	Relatively happy vs. very happy	−0.485 (0.490), *p* = 0.322	−1.330 (0.730), *p* = 0.068	−1.814 (1.160), *p* = 0.118
	Generally vs. very happy	−0.889 (0.507), *p* = 0.079	−2.389 (0.756), *p* = 0.002	−3.277 (1.200), *p* = 0.006
	Unpleasant vs. very happy	−1.618 (0.569), *p* = 0.004	−3.605 (0.864), *p* < 0.001	−5.222 (1.348), *p* < 0.001
	Very unpleasant vs. very happy	−1.493 (0.986), *p* = 0.130	−3.239 (1.304), *p* = 0.013	−4.731 (2.190), *p* = 0.031

Participation in leisure activities	Occasional participation vs. never	0.241 (0.243), *p* = 0.322	0.089 (0.373), *p* = 0.811	0.330 (0.582), *p* = 0.571
	Sometimes participation vs. never	−0.123 (0.434), *p* = 0.778	−0.840 (0.634), *p* = 0.185	−0.963 (1.023), *p* = 0.346
	Often participation vs. never	−0.316 (0.671), *p* = 0.638	−1.715 (1.184), *p* = 0.147	−2.029 (1.799), *p* = 0.259

Previous narrative training experience	Sometimes participation vs. often	−0.699 (0.402), *p* = 0.082	−0.818 (0.611), *p* = 0.181	−1.517 (0.970), *p* = 0.118
	Occasional participation vs. often	−1.172 (0.391), *p* = 0.003	−1.587 (0.602), *p* = 0.002	−3.029 (0.949), *p* = 0.001
	Never participation vs. often	−2.394 (0.444), *p* < 0.001	−3.288 (0.668), *p* < 0.001	−5.681 (1.053), *p* < 0.001

*Note:* Values are parameter estimates (B), standard errors (SEs) and *p* values from the covariate‐adjusted BCH model. The reference categories were 0 night shifts/month, very happy work mood, never participating in leisure activities and often participating in previous narrative training experience.

## 4. Discussion

This study identified four extra‐task workload profiles among clinical nurses and found that narrative cognitive ability differed significantly across profiles after covariate adjustment. These findings suggest that extra‐task workload is not a uniform experience but comprises distinct combinations of work environment demands and individual behavioural constraints among clinical nurses [11]. From the perspective of the JD–R theory, extra‐task workload can be conceptualised as an additional job demand because it requires nurses to allocate time, attention and cognitive resources beyond routine clinical responsibilities [28]. JD–R theory proposes that job demands require sustained physical or psychological effort and may consume available resources, whereas job resources help individuals manage these demands and maintain work‐related functioning [29]. Therefore, examining the combinations of demands and constraints may help explain differences in narrative cognitive ability across extra‐task workload profiles.

Rather than simply showing that clinical nurses experience additional workload, the present findings further clarify how extra‐task workload is organised within the nursing population. Work environment demands and individual behavioural constraints did not increase in parallel across the four profiles. Some nurses reported moderate demands with minimal or low constraints, whereas others showed elevated demands with moderate constraints or high levels on both dimensions. These findings suggest that how clinical nurses experience and manage additional responsibilities may be as important as the overall level of extra‐task workload. Previous studies have shown that higher nursing workload is associated with fatigue, psychological strain and adverse work‐related outcomes among nurses [30–32]. The present study extends this literature by demonstrating that clinical nurses with different combinations of additional demands and behavioural constraints may show different levels of narrative cognitive ability.

The moderate demands–minimal constraints profile had the highest narrative cognitive ability. This finding suggests that moderate additional demands are not necessarily associated with poorer professional functioning when accompanied by fewer behavioural constraints. According to the JD–R theory, job demands require sustained effort and may consume available resources when they exceed individuals’ adaptive capacity [33]. One possible explanation is that nurses in this profile had greater capacity to organise their tasks and preserve attention for patient‐centred communication and reflective practice. Conversely, the high demands–high constraints and elevated demands–moderate constraints profiles had relatively lower narrative cognitive ability, with no significant difference between the two groups. These findings suggest that the association between extra‐task workload and narrative cognitive ability may depend not only on the level of additional demands but also on the accompanying behavioural constraints. When additional responsibilities are accompanied by greater behavioural constraints, they may compete for the cognitive resources needed to understand patients’ experiences, integrate contextual information and engage in reflective nursing practice [34].

The relatively lower narrative cognitive ability observed in the elevated demands–moderate constraints profile may reflect a resource‐allocation mechanism. Additional responsibilities may consume the time and attentional resources needed for sustained patient interaction and reflective processing, whereas behavioural constraints may limit nurses’ flexibility in reprioritising tasks or adapting to competing demands. When extra‐task demands remain elevated, even moderate constraints may therefore leave limited cognitive capacity for narrative engagement. This interpretation is consistent with the JD–R framework and supports viewing extra‐task workload as a configuration of demands and control rather than as a single cumulative score [35].

The association between extra‐task workload profiles and narrative cognitive ability may also be explained by continuous narrative practice during routine clinical work. Narrative‐based approaches emphasise eliciting, interpreting and translating patients’ stories into care decisions [36]. Repeatedly switching between direct care and extra‐task duties may fragment clinical work, reducing opportunities for sustained patient interaction and reflective practice. Studies have shown that nursing interruptions are common and may disrupt task continuity and clinical performance [37, 38]. Consequently, nurses in profiles characterised by higher demands or greater behavioural constraints may have fewer opportunities to engage deeply with patients’ illness narratives, which may help explain the observed differences in narrative cognitive ability.

After covariate adjustment, work‐related mood and narrative training experience were significantly associated with narrative cognitive ability. This is consistent with previous evidence that nurses’ work‐related well‐being and narrative‐related education are associated with narrative competence and engagement in narrative practice [39]. A positive work‐related emotional state may facilitate the empathy and reflective orientation required for narrative care, whereas narrative training may provide the conceptual knowledge and practical skills needed to engage with patients’ stories [40]. Differences in narrative cognitive ability across extra‐task workload profiles remained significant after controlling for these factors, suggesting that the combination of work environment demands and behavioural constraints may represent an additional correlate beyond individual affective and training‐related factors. Conversely, monthly night‐shift frequency and participation in leisure activities were not significantly associated with narrative cognitive ability after adjustment. Monthly night‐shift frequency may not adequately capture the actual burden of night work, as nurses with a similar number of night shifts may experience different levels of sleep disruption and cumulative fatigue, which may influence the cognitive resources available for processing and reflecting on clinical experiences [41]. Similarly, leisure activities may facilitate recovery by promoting psychological detachment from work, but their potential benefits may depend more on their restorative quality than on participation frequency alone. Future studies should use more comprehensive measures of sleep, fatigue and leisure‐related recovery to clarify these associations.

Overall, extra‐task workload among clinical nurses appears to be a multidimensional and heterogeneous work experience rather than a uniform increase in workload. Interpreted within the JD–R framework, the identified combinations of work environment demands and behavioural constraints highlight the importance of considering both dimensions when examining how additional responsibilities are associated with clinical nurses’ professional functioning. Given the cross‐sectional design, these associations should not be interpreted as causal relationships and require confirmation in longitudinal and multicentre studies.

### 4.1. Implications for Nursing Management

The identified workload profiles provide a basis for profile‐informed management strategies rather than uniform interventions. Nursing managers may incorporate assessments of work environment demands and behavioural constraints into routine workload monitoring to identify nurses requiring different levels of support.

For nurses in the high demands–high constraints profile, management priorities should focus on reducing excessive workload exposure and increasing control over additional responsibilities. Managers may review non–direct‐care tasks, remove unnecessary administrative duties, clarify role boundaries and strengthen supervisory support. These strategies may improve workflow organisation and ensure that additional duties are allocated appropriately [42].

For nurses in the elevated demands–moderate constraints profile, which represented the largest subgroup, prevention of cumulative workload burden may be particularly important. Regular review and transparent allocation of teaching, research, quality improvement responsibilities and other additional duties may help maintain manageable workload levels.

Beyond workload optimisation, role clarification and professional development may provide additional support. Clear role descriptions, structured task allocation and effective team communication may reduce ambiguity surrounding extra‐role duties and improve coordination [43]. Additionally, narrative training may support nurses in more demanding workload profiles. Practice‐based approaches, such as reflective discussion and case‐based narrative learning, may be integrated into existing professional development programmes to strengthen narrative practice [44].

### 4.2. Limitations

This study has several limitations. First, the cross‐sectional design precludes causal inference; therefore, the temporal relationship between extra‐task workload profiles and narrative cognitive ability should be examined in longitudinal studies. Second, participants were recruited by convenience sampling from a single tertiary hospital in Henan Province, China. The relatively homogeneous sample in terms of gender, educational level and occupational characteristics may limit the generalisability of the identified profiles to other regions, hospital settings and nursing populations. In addition, institution‐specific practices in the organisation and allocation of training, administrative duties and other extra‐task activities may shape nurses’ extra‐task workload patterns and, consequently, the profiles identified in this study. Therefore, the profile structure and distribution observed in this hospital may not be directly generalisable to institutions with different task‐management practices. Third, data were collected using self‐reported questionnaires at a single time point. Although procedural remedies and Harman’s single‐factor test suggested that common method bias was unlikely to have substantially influenced the findings, residual bias cannot be completely excluded. Future multicentre studies incorporating longitudinal designs and multisource data are warranted.

## 5. Conclusions

This study identified four extra‐task workload profiles among clinical nurses based on work environment demands and individual behavioural constraints. Narrative cognitive ability differed significantly across the profiles, indicating that extra‐task workload is a heterogeneous experience among clinical nurses. A person‐centred approach may help nursing managers identify different support needs and develop profile‐specific strategies for workload management, role clarification and professional development. Future longitudinal and intervention studies are needed to validate these associations and evaluate profile‐specific management approaches.

## Author Contributions

Xue Li: conceptualisation, formal analysis, investigation, data curation, writing–original draft and writing–review and editing.

Weihong Yang: supervision, data collection and writing–review and editing.

Ling Guo, Ruijuan Fan and Hua Peng: investigation, data collection and quality control.

Fengpei Zhang: study design, methodology, supervision, resources and writing–review and editing.

## Funding

This study was supported by the Henan Province Medical Science and Technology Research Project (grant number: LHGJ20230495) and the ‘Li Xiuzhen Youth Scientific Research Fund’ Project of the First Affiliated Hospital of Henan Medical University (grant number: HLQN‐2025‐A02). These funds supported the conduct of the research, including data collection and other study‐related activities.

## Disclosure

The funders had no role in the study design, data analysis, interpretation of the findings or preparation of the manuscript.

## Ethics Statement

This study was approved by the Ethics Committee of the First Affiliated Hospital of Henan Medical University, China (Ethics review number: EC‐025‐591). Electronic informed consent was obtained from all participants before questionnaire completion. The online questionnaire was administered anonymously, and participant confidentiality was ensured throughout the study.

## Conflicts of Interest

The authors declare no conflicts of interest.

## Data Availability

The anonymised dataset used and/or analysed during the current study is available from the corresponding author upon reasonable request, subject to ethical and institutional requirements.
